# Foreign Body in the Urinary Bladder: Another Outpatient Presentation

**DOI:** 10.7759/cureus.27064

**Published:** 2022-07-20

**Authors:** Okelue E Okobi, Imolikhe C Imobighe, Chukwudike G Nnaji, Ngozi J Adaralegbe, Zainab Akinsola, Endurance O Evbayekha

**Affiliations:** 1 Family Medicine, Lakeside Medical Center, Belle Glade, USA; 2 Obstetrics and Gynecology, Irrua Specialist Teaching Hospital, Irrua, NGA; 3 Internal Medicine/Family Medicine, Windsor University School of Medicine, Chicago, USA; 4 Allied Health Sciences, University of Connecticut, Waterbury, USA; 5 Internal Medicine/Family Medicine, Windsor University School of Medicine, Toronto, CAN; 6 Internal Medicine, St. Luke's Hospital, St. Louis, USA

**Keywords:** urinary bladder, primary care physicians, retained foreign body, foreign body removal, foreign body in the bladder

## Abstract

Primary care physicians manage a variety of medical conditions in their practice; to do this successfully, they require proper preparation and a wealth of experience, which they garner over years of practice. This case describes a 41-year-old male who presented to an outpatient clinic with a foreign body in the urinary bladder. This case report captures the level of preparedness required in primary care, the challenges that come with the broad spectrum of disease presentations, inter-specialty collaboration, and consultation or referral as appropriate with regards to having a foreign body in the urinary bladder. This report also shows how misinformation from patients can play a part in delayed diagnosis of simple presentations such as a self-inserted foreign body.

## Introduction

In primary care, foreign bodies in the lower urinary tract may be encountered. However, this common presentation, which requires a simple standard ambulatory approach to diagnosis and collaborative management, may be complicated by several factors, including the level of preparedness in a facility, misinformation, and the complexities of patients' autonomy. Primary care is defined by the American Academy of Family Physicians (AAFP) as "the provision of integrated, accessible health care services by physicians and their health care teams, who are accountable for addressing the vast majority of health care needs, developing a sustained partnership with patients, and practicing in the context of family and community." It is person-centered, team-based, community-aligned, and designed to achieve better health care and lower costs [1]. This definition captures the level of preparedness required in primary care practice to confront the daily challenges of the spectrum of disease presentations that can range from typical medical to surgical presentations, from pre-conception to their entire life span, as well as inter-specialty collaboration and the use of consultation or referral as appropriate. However, most primary care practices continue to face the challenge of limited resources (equipment, trained personnel, supplies) [2-4]. This clinical case scenario exemplifies the complex skills and inter-specialty collaboration necessary to provide care. Furthermore, this case demonstrates another addition to the documented cases of a foreign body in the bladder with a twist of misinformation that turned out to be a foreign body in the urinary bladder. It also reiterates multidisciplinary collaboration in addressing common problems. The management of foreign bodies in the urinary bladder has been reported to require a basic medical history from patients to direct physical examination, investigation, and removal procedures. Incomplete histories can pose diagnostic challenges and frequently result in "over-investigation," resulting in increased use of healthcare resources and costs.

## Case presentation

A 41-year-old man presented to our outpatient clinic with complaints of two days of mild rectal pain, suprapubic pain, dysuria, and a history of self-insertion of a tennis ball into his rectum for an undisclosed reason. The patient denied any history of abuse, psychiatric disorder, or trauma. An examination revealed a stable patient with capacity and normal mental state examination findings. Furthermore, physical examination findings were normal but for mild suprapubic tenderness; otherwise, other abdominal examination findings were normal. His digital rectal examination was normal. Sigmoidoscopy was done post-digital rectal examination and was negative.

**Figure 1:**
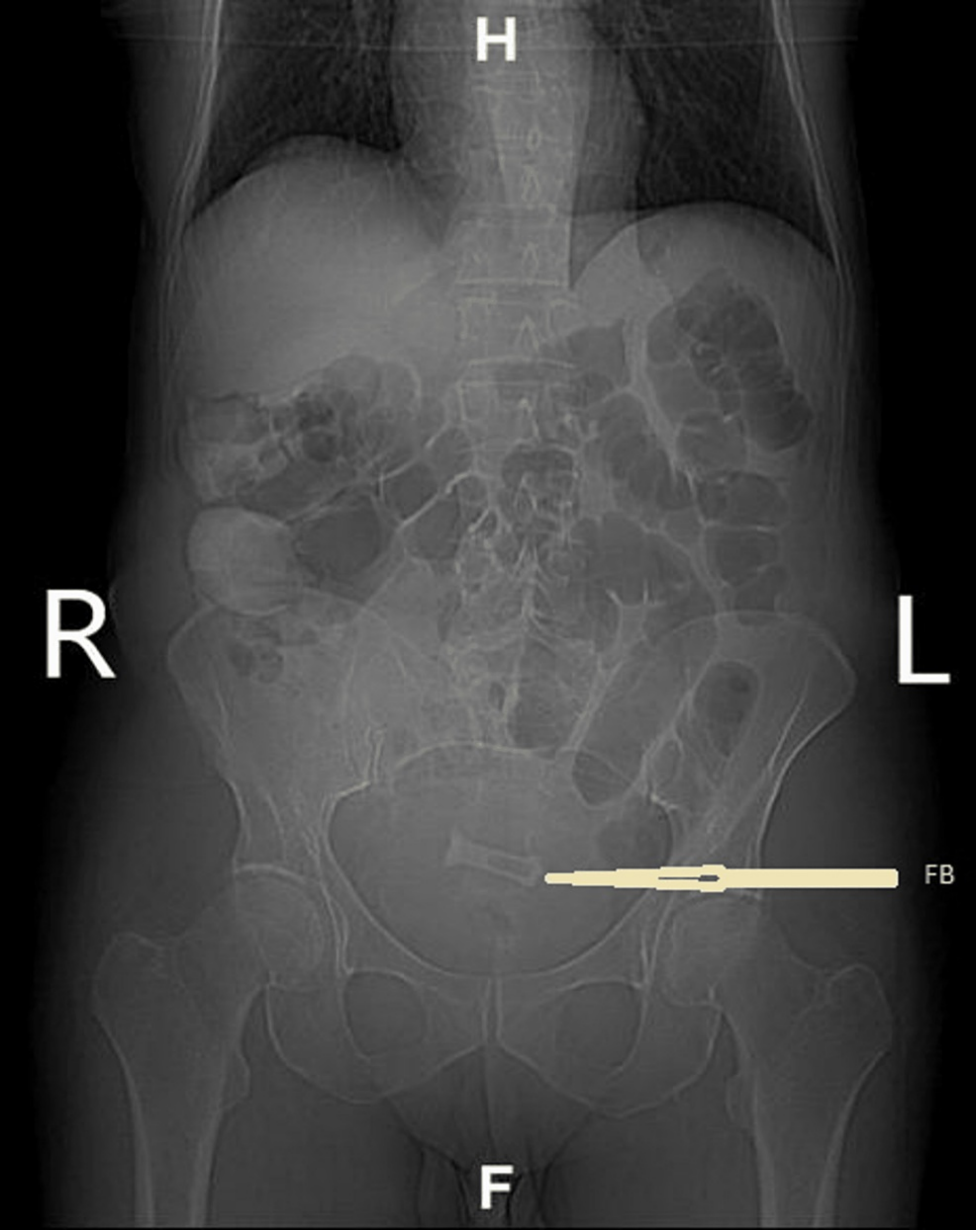
Arrow pointing to a foreign body (FB) in the urinary bladder

**Figure 2:**
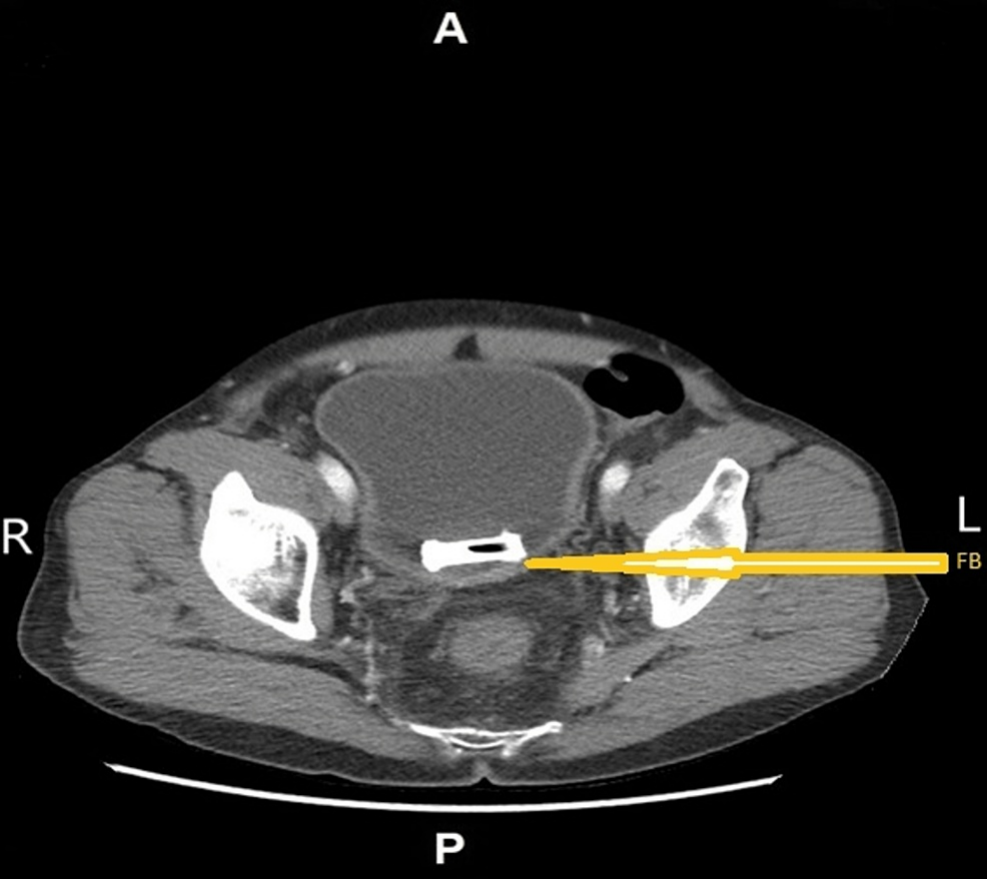
Arrow pointing to a foreign body (FB) in the urinary bladder

Apart from the preponderance of epithelial cells in the urinalysis, it was negative for other dipstick indicators of urinary tract infection (UTI). An abdominal X-ray followed by a KUB (X-ray specific for kidneys, ureters, and urinary bladder) revealed a radiopaque body, suggestive of a tiny tube in the pelvic midline (Figure 1). A CT scan revealed an approximately 2x3 cm amorphous metal-like foreign body (FB) within the urinary bladder with mild bladder wall thickening (Figure 2). The patient was managed prophylactically for UTI and referred to a urologist for further FB evaluation and removal options. Upon referral, the patient declined cystoscopy and other work-up or foreign body removal procedures for personal reasons. The patient subsequently returned to the clinic and was counseled on the dangers of foreign bodies in situ, risks, and complications. He declined questions concerning the method of insertion or length of FB in the body. The patient was discharged in stable condition and was scheduled for a follow-up with a psychiatrist for further evaluation and a urologist for further care and monitoring.

## Discussion

Physicians and their health care teams are responsible for providing integrated and accessible health care, developing a lasting partnership with patients, and practicing in the context of family, community, person-centered, team-based, and community-aligned care that improves health care and costs. They carry out their responsibilities while facing resource limitations like a shrinking primary care workforce, skills sets, and training gaps. Oftentimes, they are faced with a common illness that may present in an uncommon manner. Cases of foreign bodies in the urinary bladder are such examples. Diagnosing and managing foreign bodies in the urinary bladder requires a patient-centered medical history, targeted physical examination, appropriate investigation, and foreign body removal procedures as needed. Incomplete history often leads to "over investigation" and increased healthcare costs. Some authors have documented several cases involving a foreign body in the urinary bladder that has been recorded [5-8]. The diagnosis and treatment of foreign bodies in the bladder in an outpatient setting can be challenging but can be managed via a wide range of approaches.A simple standard ambulatory approach to diagnosis may be sufficient in stable patients. The diagnosis is supported by good medical history, focused physical examination, and investigation [5-7]. Common presenting symptoms include fever, symptoms of cystitis, UTI (dysuria, hematuria, frequency), and, as seen in this case report, pain (suprapubic). However, sometimes, patients may be asymptomatic. In instances of self-insertion for sexual gratification or other related patient reasons, psychiatric disorders like dementia, drug intoxication, or associated conditions, a high index of suspicion is often required, as seen in our case scenario. Several retrospective studies have documented that lower urinary tract foreign bodies can be caused by self-infliction, migratory, iatrogenic causes, sexual abuse, psychiatric illnesses, or other rare causes [6,8-14]. Some authors report varying percentages in the incidence of the entry route [6-14]. The true incidence of FBs may vary. For instance, that of retained foreign objects is not known. Still, there is a reported incidence of about 1 in 100 to 5000 of all surgical interventions and 1 in 1000 to 1500 for all intra-abdominal operations [6,8-15]. Common investigations, like bedside ultrasound scans, may reveal the position of these objects. These are commonly done in conjunction with urinalysis and the assessment of kidney function. Following diagnosis, retrieving foreign bodies from the urinary bladder in an outpatient setting may pose a significant challenge. A common approach is a referral to a competent level of care (emergency department or a urologist) where complications that may result from the removal process can be adequately handled. The AAFP requires primary physicians to determine when to provide ethical medical care and when to refer to other more competent collaborating physicians [15-17]. Furthermore, in the management approach for urinary bladder FB, when further investigation is warranted, a cystoscope for visualization and determination of the nature, position, and size of the FB will help guide the method of removal and is frequently recommended [6-14]. Endoscopy and minimally invasive techniques are common management approaches [7-14]. In other severe cases, open surgery may be an option [5-14,18]. Certain circumstances may complicate these sequential modalities and necessitate a broader investigation [7-10]. Objects that have been retrieved include pens, pencils, thermometers, intrauterine devices, needles, self-made magnetic pipes, fishbones, bullets, screws, infant feeding tubes, etc. [6-14]. Complications of unremoved FB may include intermittent urinary obstruction depending on the size and position of the object, cystitis, UTI, encrusting and possible stone formation, bladder wall erosion, penetration, and fistulation. Bladder infections due to a foreign body often necessitate removal, and the complication of bladder wall perforation was found in some cases [6-14]. Given the plethora of complications that patients develop when foreign bodies are left in situ, the never-ending debate about when patients' autonomy conflicts with the professional need to practice beneficence continues to reverberate in today's medical world. However, medical practice favors autonomy legally and ethically, especially when capacity has been determined. Nevertheless, sometimes, patients' autonomy may lead to harm; unfortunately, "harm" may be difficult to define in some cases as it may vary from person to person, even in those with capacity. In this case study, the well-known medical approach to patient management about shared-collaborative effort between patient and physician in a mutually respectful alliance was adopted. This approach is echoed in the American Medical Association's code of medical ethics that "patients are to make decisions about the care the physician recommends and to have those decisions respected; for example, a patient with decision-making capacity may accept or refuse any recommended medical intervention."

## Conclusions

Cases of FB in the lower urinary tract system will continue to present in our clinics. In some cases, outpatient management approaches may be challenging. In most primary care settings, foreign bodies in the urinary bladder are diagnosed and retrieved with minimal systemic involvement; however, it often requires multidisciplinary collaboration. Incomplete or poor history usually leads to a wild goose chase; hence, a high index of suspicion is necessary for proper management. Incomplete history may arise for different reasons, including an intention to conceal, mainly when applied for perceived therapeutic reasons, autoerotic, fear of privacy breach, psychiatric, or for other undefined reasons by the patient. Physicians then need to utilize skills gained from years of practice to balance the intricate relationship between patient autonomy and quality care to get the necessary information from the patients.
